# Immunolocalization of Na(+)-Dependent Glucose Co-Transporters in Chicken Kidneys in Norm and During T-2 Mycotoxicosis (Primary Study)

**DOI:** 10.3390/cimb46120854

**Published:** 2024-12-18

**Authors:** Cristin Allmang, Piret Hussar, Ilmārs Dūrītis, Florina Popovska-Percinic

**Affiliations:** 1 Institute of Veterinary Medicine and Animal Sciences, Estonian University of Life Sciences, 51006 Tartu, Estonia; cristin.allmang@emu.ee; 2 Faculty of Medicine, University of Tartu, 50411 Tartu, Estonia; 3 Faculty of Veterinary Medicine, Latvian University of Agriculture, LV-3004 Jelgava, Latvia; ilmars.duritis@llu.lv; 4 Faculty of Veterinary Medicine, Ss. Cyril & Methodius University in Skopje, 1000 Skopje, North Macedonia; florinap@fvm.ukim.edu.mk

**Keywords:** chicken, immunohistochemistry, sodium-dependent glucose co-transporter, mycotoxicosis

## Abstract

The kidney plays an essential role in the proper homeostasis of glucose. In the kidney, glucose transport is carried out across cell membranes by two families of glucose transporters—facilitated diffusion glucose transporters (GLUTs) and Na(+)-dependent glucose co-transporters (SGLT family). Among the transporters, sodium-dependent glucose co-transporters play a major role in the kidney‘s ability to reabsorb glucose. Although the localization of glucose transporters has been extensively studied in mammals, there are still knowledge gaps regarding the localization of SGLTs in birds. The aim of this research was to conduct a comparative study of the immunolocalization of the sodium-dependent glucose co-transporters SGLT1 and SGLT2 in the kidneys of healthy and T-2-mycotoxicated chickens. Immunohistochemical staining was carried out using the polyclonal primary antibodies SGLT1 and SGLT2 (Abcam, UK) in kidney tissue derived from seven healthy and seven T-2-mycotoxicated 7-day-old female layer-type Ross chickens (*Gallus gallus domesticus*). The sections were stained using an immunohistochemistry kit (Abcam, UK). In the kidneys of the healthy birds, strong staining of SGLT1 and SGLT2 was observed in the cytoplasm of the epithelial cells of the proximal straight and convoluted tubules. In the kidneys of the birds of the T-2 toxin group, weak expression of SGLT1 and SGLT2 with morphological changes occurred, indicating reduced glucose transport in the urinary system during T-2 mycotoxicosis.

## 1. Introduction

**Figure 1:**
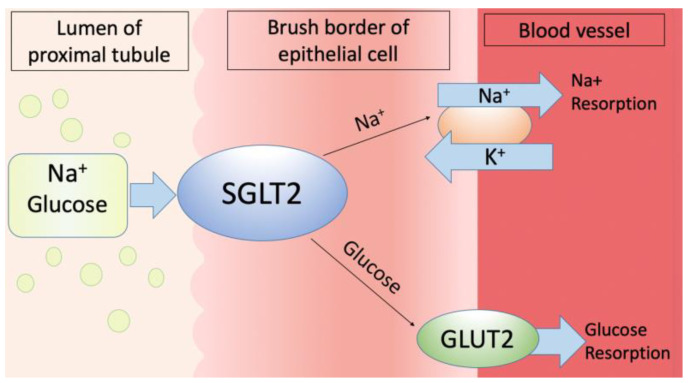
Na(+)-dependent glucose co-transporter 2 transmembrane transport in bird kidneys. SGLT2 = Na(+)-dependent glucose co-transporter 2; GLUT2 = facilitative glucose transport.

As glucose is the primary source of energy for all living organisms, its homeostasis is particularly important [1]. The kidneys play a major role in maintaining the body’s glucose homeostasis, not only in filtering but also in absorbing the glucose [2]. The primary filtration unit in the kidneys, the glomerulus, filters the glucose, which is then reabsorbed in the kidney’s proximal convoluted tubule by the sodium-dependent glucose co-transporters SGLT1 and SGLT2, as the glucose transport across the apical brush border of renal epithelial cells requires the presence of a sodium gradient [3,4,5,6]. The glucose transport across the cell membrane is mediated by two families of glucose transporters: the facilitated diffusion glucose transporters (GLUTs) and the Na(+)-dependent glucose co-transporters (SGLT family) [7]. GLUTs and SGLTs vary in their substrate specificity and regulatory mechanisms, as well as in their distribution [8]. While the GLUTs transport glucose across the plasma membrane via the mechanism of facilitated diffusion, the sodium-dependent glucose co-transporters belong to the family of active glucose transporters, which simultaneously transport sodium and glucose using a concentration gradient [9]. A low intracellular sodium concentration is created and maintained by the basolaterally located sodium–potassium–adenosine triphosphatase pump, which forces intracellular sodium out of the cell through the basolateral membrane; then, the electrochemical gradient provides the driving force for the transport of sodium into the cell across the apical membrane and for the glucose transport by specific sodium-dependent glucose transporters (SGLTs) [10]. When glucose is concentrated in epithelial cells to a level that exceeds the interstitial glucose level, it diffuses into the interstitium through specific facilitative glucose transporters located on the basolateral membrane. The facilitative glucose transporter GLUT2 mediates glucose transport across the basolateral membrane along its chemical gradient. Basolateral GLUT1 may help to reabsorb glucose or take up glucose from the peritubular space. Figure 1 represents the mechanism of transmembrane glucose transport by SGLT2, which is situated at the brush border of epithelial cells [11].

Since SGLT2 carries out 80–90% of glomerular filtered glucose reabsorption, it is considered the main co-transporter involved in glucose reabsorption in the kidney [12,13]. The remainder of the glucose absorption, which is approximately 10%, is carried out by SGLT1 [4,14]. The localization of glucose transporters in the gastrointestinal tract of birds has previously been extensively studied, and these studies have shown differences in glucose transporter immunohistochemical staining in different age groups of laying chickens and ostriches [15,16]. However, the immunolocalization of sodium-dependent glucose co-transporters in the kidney tissue of birds has only recently begun [11,16,17]. Because the kidneys of avians possess different types of glomeruli—the mammalian type and the reptilian glomeruli type [3]—they differ significantly from mammalian kidneys.

Mycotoxins, which are toxic secondary metabolites, are produced by filamentous fungi belonging to the phylum Ascomycota or by molds; they have great importance in the health of humans and animals as they are the cause of acute and chronic diseases [18]. Of the approximately 400 mycotoxins, 30 are considered to be a threat to human or animal health; among these, aflatoxins, ochratoxins, fumonisins, and trichothecenes are the most important. Trichothecenes are groups of mycotoxins that are produced by diverse filamentous fungal species, including *Fusarium*, *Myrothecium*, *Stachybotrys*, *Trichoderma*, *Trichothecium*, and *Spicellum* [19,20,21]. Among mycotoxins, T-2 toxin is the most toxic trichothecene, and it is produced by different *Fusarium* species, including *F. sporotrichioides*, *F. poae*, and *F. acuminatum*. The presence of these *Fusarium* species has been documented to occur in moderate to cold climates and wet storage conditions [22,23]. *F. sporotrichioides* is the main T-2 producer, and it is able to grow in a wide range of temperatures, from −2 to 35 °C [24]. The toxins produced by T-2 are a significant threat to human and animal health. They can increase susceptibility to infections by immunosuppression and are able to cause necrosis in the digestive tract and dystrophy in other organs, such as the liver, kidneys, heart, brain, or the peripheral ganglia of the vegetative nervous system [25,26,27]. In poultry, the consumption of feed contaminated with T-2 reduces the chicken’s weight gain, egg production, and hatching ability [28]. It is hypothesized that the T-2 toxin inhibits protein synthesis through the binding and inactivation of peptidyl-transferase at the site of DNA transcription [29,30]. The main sources of trichothecenes include contaminated wheat, barley, rye, oats, and maize; however, they also occur in hay, straw, and green feed, as well as silage from contaminated cereals [26,31,32,33]. Trichothecenes can enter the human food chain via the consumption of products of animal origin, such as meat, milk, and eggs, that are derived from livestock fed with trichothecene-contaminated feed [31,34,35]. Although the localization of the sodium-dependent glucose co-transporters is relatively well established in mammal kidney tissue, the same comprehensive scientific studies on the immunolocalization of SGLT1 and SGLT2 in the kidneys of healthy and diseased birds are lacking.

As there is a current need for comparative studies on the effect of T-2 mycotoxin on the expression of glucose transporters, specifically sodium-dependent glucose co-transporters-1 (SGLT1) and -2 (SGLT2), in avian kidneys, the aim of our experimental investigation was to immunolocalize the sodium-dependent glucose co-transporters-1 and -2 and to compare them in healthy and T-2-intoxicated laying-type chickens.

## 2. Materials and Methods

Kidney material was collected from seven 7-day-old healthy broilers (control group) and seven 7-day-old female layer-type broilers with T-2 toxicosis (T-2 toxin group). The Ross broilers (*Gallus gallus domesticus*), obtained from a commercial Macedonian hatchery, were placed in temperature-controlled brooders and raised in standard conditions with free access to food and water. For the T-2 toxin group, starting from the fourth day after hatching, T-2 toxin was applied at a dose of 0.250 mg/day/bird and was compulsory for three consecutive days. Twenty-four hours after the last dosage of the T-2 mycotoxin, the chickens were sacrificed with an intracardiac overdose of 0.5 mL 20% sodium pentobarbital, and the kidneys were removed. Specimens of 0.5–1.0 cm in diameter were fixed in 10% buffered formalin solution for at least 72 h to ensure complete fixation. Thereafter, the specimens were embedded in paraffin, and 7 μm thick slices were cut. The prepared slides were stained using the standard hematoxylin and eosin staining method [36] and immunohistochemical staining with the polyclonal primary antibodies rabbit anti-SGLT-1 and rabbit anti-SGLT-2 (Abcam, Cambridge, UK). To immunolocalize SGLT1 and SGLT2 in the chicken kidney tissue, the sections were stained using an immunohistochemistry kit (Abcam, UK) containing the secondary antibody, according to the manufacturer’s guidelines (IHC kit, Abcam, UK). The sections were pre-treated using heat-mediated antigen retrieval with sodium citrate buffer (pH 6) for 20 min; then, they were incubated with the primary rabbit polyclonal antibodies rabbit anti-SGLT1 and rabbit anti-SGLT2 (Abcam, UK) at a 1:1000 dilution for 30 min at 37 °C. Biotinylated secondary antibody at a 1:1000 dilution and streptavidin-conjugated peroxidase were used for detection, using DAB as a chromogen. The negative controls contained antibody diluent (Dako, S0809, Glostrup, Denmark) instead of primary antibodies, and ostrich chicken kidney tissue sections were used as positive controls to identify SGLT1 and SGLT2. The IHC study was performed twice to confirm the results of the first experiment.

The immunolocalization of SGLT1 and SGLT2 was studied by two scientists using an eye visual control in a blind analysis, and the intensity of SGLT1 and SGLT2 expression in the proximal tubules of chicken kidneys were categorized as weak (+), moderate (++), or strong (+++). The photos of the slides were taken using a PreciPoint M8 digital microscope (PreciPoint, Garching bei München, Germany), in magnification 400×.

The statistical analysis was performed using Python software (version 3.12.4, 2024). To compare the control and T-2 toxin groups, paired *t*-tests and Wilcoxon signed-rank tests were conducted.

The ethical committee of Ss. Cyril and Methodius University in Skopje, in conformity with the recommendation provided in the European Convention for the Protection of Vertebrate Animals used for Experimental and Other Scientific Purposes (ETS no. 123, Approval No. 03-7534, 12 April 2013), approved the husbandry and experimental procedures of the study.

## 3. Results

### 3.1. Routine Histology

**Figure 2:**
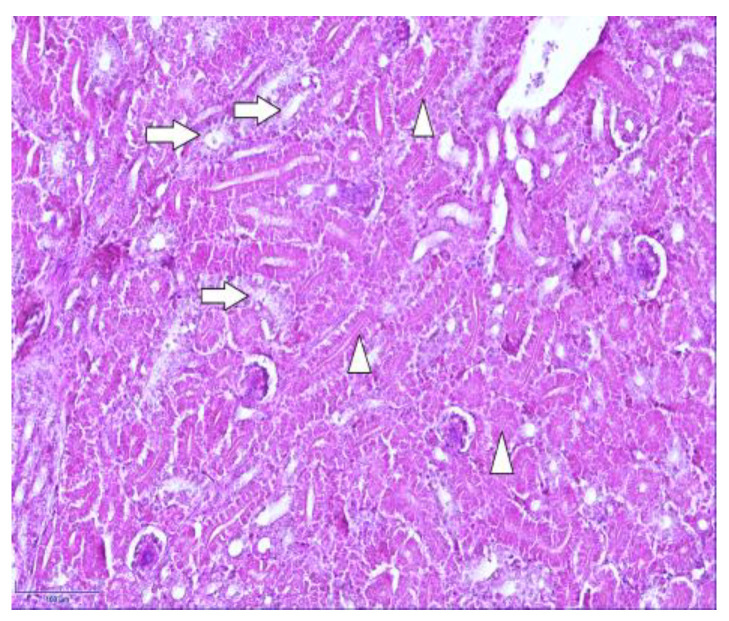
Normal kidney morphology of a 7-day-old chicken: proximal (arrowheads) and distal tubules (arrows) in the cortex of the kidney. Hematoxylin and eosin. Magnification 400×, scale bar 100 µm.

To facilitate an understanding of the normal chicken kidneys, routine histology staining using hematoxylin and eosin was carried out (Figure 2). In Figure 2, the identified proximal and distal renal tubules can be observed.

### 3.2. Immunohistochemistry

**Figure 3:**
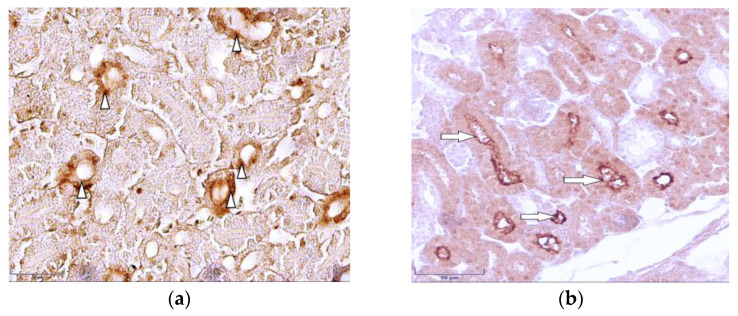
Immunolocalization of the sodium-dependent glucose co-transporter-1 (SGLT1) in kidney tissue (**a**) in healthy 7-day-old chickens; note the strong expression of SGLT1 in the apical part of the epithelial cells of renal proximal tubules (arrowheads). Magnification 400×, scale bar 50 µm; (**b**) damaged brush border membranes of proximal tubule’s epithelial cells in intoxicated chicken (arrows). Magnification 400×, scale bar 50 µm.

**Figure 4:**
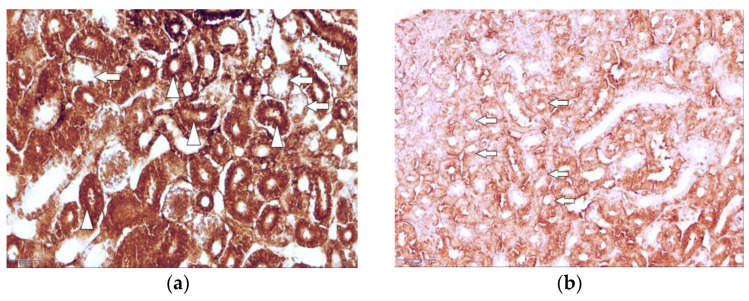
Immunolocalization of sodium-dependent glucose co-transporter-2 (SGLT2) in (**a**) strongly stained proximal tubules (arrowheads) of healthy chicken kidney and weakly stained distal tubules (arrows) is observed. Magnification 400×, scale bar 50 µm; (**b**) the pale staining of proximal renal tubules (arrows) of intoxicated bird group is observed. Magnification 400×, scale bar 50 µm.

The results of the immunohistochemical study revealed the immunolocalization of the major glucose transporters in the kidney, SGLT1 and SGLT2, which were observed in the proximal renal tubules of the hen chickens in both study groups (Figure 3a and Figure 4a). In the group of healthy chickens, a strong expression of SGLT1 occurred in the apical part of the epithelial cells of the renal proximal tubules (Figure 3a).

Compared to the healthy birds, the expression of both of the studied antibodies was weaker in the T-2 toxin group, and the brush border membranes of the proximal tubule’s epithelial cells were irregular and damaged (Figure 3b and Figure 4b).

**Figure 5:**
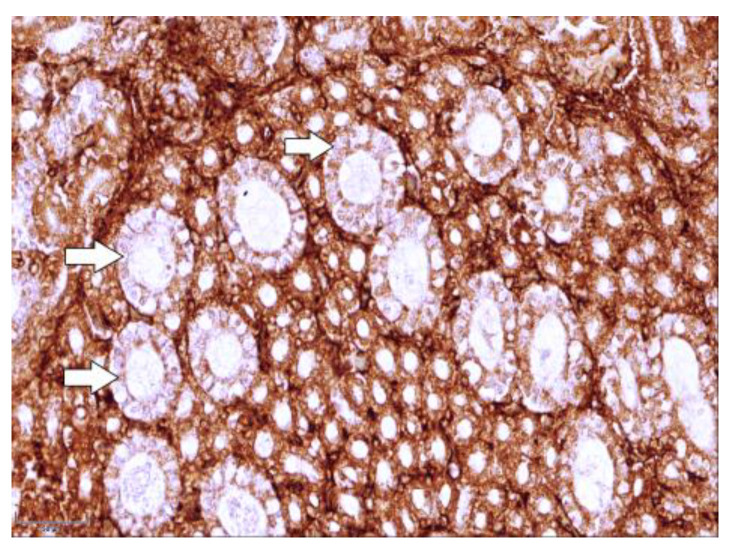
SGLT2 in healthy 7-day-old chicken kidneys. Note the unstained collecting ducts (arrows); immunohistochemistry (IHC) magnification 400×, scale bar 50 µm.

In both studied groups, no specific staining for SGLT1 and SGLT2 was observed in the distal tubules and collecting ducts of the birds’ kidneys (Figure 5).

In the comparison of both of the studied antibodies, the staining of the renal tissue of the control group was more intense than that of the T-2 toxin group. The results of the repetitive IHC studies confirmed the results of the primary experiment. Furthermore, the expression of SGLT2 was observed to be much stronger than the staining of SGLT1. The results of the descriptive (eye visual) analysis of the expression of SGLT1 and SGLT2 in the proximal tubules, which was determined according to the intensity of the staining, are shown in Table 1.

### 3.3. Statistical Analysis

To evaluate the impact of the T-2 mycotoxin exposure on the glucose transporters expression the statistical analysis was performed using software Python (version 3.12.4, 2024) which enables to convert the qualitative data obtained from the IHC study to quantitative form. The numeric values (Table 1) were converted to the intensity of staining: + = 1 (weak), ++ = 2 (moderate), +++ = 3 (strong), and the statistics were calculated for each group. During the analysis, the mean staining intensity for each group as well as the variability of the staining intensity (standard deviation = SD within each group) was found (Table 2). The results showed a significant reduction in staining intensity for both SGLT1 and SGLT2 in the T-2 toxin groups compared to the control groups. The statistical tests (paired *t*-test and Wilcoxon signed-rank test) confirmed that these differences were significant, with *p*-values less than 0.05. The variability in staining intensity, as indicated by the SD values, is slightly higher in the T-2 toxin groups, particularly for SGLT2. These findings suggest that T-2 toxin exposure significantly reduces the expression of SGLT1 and SGLT2, as evidenced by the decreased IHC staining intensity in the T-2 toxin groups.

## 4. Discussion

In maintaining the body’s homeostasis, the role of the kidneys is significant. Histologically, kidneys consist of two zones—the cortex and the medulla. The morphofunctional unit of the kidney is the nephron. Functionally, nephrons consist of glomeruli and tubules. Nephrons are responsible for filtering the blood plasma to eliminate waste products, as well as to conserve glucose and water. In birds, the homeostasis of fluid and ions needs the proper functioning of several organ systems and is a more complex phenomenon than in other vertebrates [37]. Unlike those of mammals, the kidneys of chickens contain different types of glomeruli, all of which are seen in the cortical region: a small reptilian type of glomerulus near the surface of the cortex; a larger, mammalian type of glomerulus near the medulla; and an intermediate type of glomerulus in the deeper cortex regions. The kidney maintains glucose homeostasis by reabsorption of glucose in the proximal tubules, the release of glucose into the circulation via gluconeogenesis, and the uptake of glucose from the circulatory system to provide itself with energy [6]. Glomeruli are the primary filtration units because they filter glucose from plasma, which is then reabsorbed through glucose transporter proteins that are found in the cell membranes within the proximal tubules of the kidney. The proximal renal tubule, which is a part of the nephron, can be further divided into two sections: the proximal convoluted tubule and the proximal straight tubule. These two sections are subdivided into ultrastructural divisions, known as segments, which consist either of higher or lower cell complexity. While the S1 segment corresponds to the convoluted proximal tubule, the S2 and S3 segments correspond to the straight proximal tubule [38]. The studies on mammalian renal tissue have shown that the kidney’s proximal tubules contain the necessary enzymes for gluconeogenesis (lactate, glutamine, glycerol, and alanine), as well as the main transporters that are active in the process of glucose reabsorption—the Na(+)-dependent glucose co-transporters SGLT2 and SGLT1 on the apical membrane and the facilitated diffusion glucose transporter GLUT2 on the basolateral membrane. The glucose reabsorbed or produced in the proximal tubule (gluconeogenesis) is mainly absorbed in the peritubular capillaries and returned to the systemic circulation or used as an energy source in the distal tubule segments, which take up glucose via basolateral GLUT1. The SGLTs are considered active transporters, and the GLUTs belong to the family of passive transporters [39]. In our previous study, the immunolocalization of SGLT1 and SGLT2 was revealed in the kidneys of chickens of different ages [40]. For SGLT1, strong staining in the apical parts of the epithelial cells of the straight proximal tubules was observed, and for SGLT2, strong staining of the renal proximal tubules and unstained distal tubules was observed in the kidneys of laying chickens of different ages, showing that the staining was found to be similar among all different age groups. The immunohistochemical localization of SGLT1 and SGLT2 in the kidneys of hen chickens of different ages was detected on the apical side of the epithelial cells of the proximal renal tubules. The findings in the previous studies on the localization of the sodium-dependent glucose co-transporters SGLT1 and SGLT2 in the renal tissue of chickens of different age groups are in accordance with the findings of our current study on young laying chickens, where the immunolocalization of SGLT1 and SGLT2 was detected on the apical membrane of the epithelial cells of the chicken kidney’s proximal tubules.

Mycotoxins are low-molecular-weight secondary metabolites of fungi [41]. Mycotoxins can be divided according to the organ affected, such as nephrotoxic, hepatotoxic, or immunotoxic, or into general groups, such as allergens, teratogens, or carcinogens [42]. The T-2 toxin studied in our current experiment is a carcinogenic mycotoxin that belongs to the group of *Fusarium* mycotoxins in the trichothecene family and is widely encountered as a natural contaminant that is known to elicit toxin responses in different organs and tissues [43]. Mycotoxins, which are secondary metabolites produced by several fungal species, can exert severe toxic effects on humans and animals [44]. In the trichothecene family, the T-2 mycotoxin is amongst the most toxic members when compared to other mycotoxins, causing serous hemorrhagic inflammation, necrosis and ulceration in the digestive tract, and dystrophy in the kidney, liver, heart, brain, and peripheral ganglia of the vegetative nervous system. Additionally, T-2 has been shown to reduce egg production and weight gain in chickens and to impede egg hatching ability [45]. In poultry, the T-2 toxin has also been observed to be the causative agent of the impairment of immune responses, the destruction of the hematopoietic system, declining egg production, serous-hemorrhagic necrotic–ulcerative inflammation of the digestive tract, internal hemorrhaging, and mouth and skin lesions [44]. Pathohistological studies usually reveal fatty changes and strong granular degeneration, mainly in the kidneys and liver. In chronically T-2-intoxicated poultry, interstitial nephritis, glomerunephritis, and kidney sclerosis are among the main pathologies that can be observed [46].

The mechanism of action of the T-2 mycotoxin primarily functions by inhibiting protein synthesis, disrupting nucleic acids, and inducing oxidative stress generating free radicals. The thiol group of the T-2 toxin makes it a potent DNA synthesis and protein inhibitor and it even reduces lymphocyte proliferation. T-2 has the ability to impair the production of antibodies and alter the membrane function of dendritic cells [45]. While its impact on renal glucose transporters SGLT1 and SGLT2 is not specifically detailed in existing literature, the broader mechanisms of T-2 toxicity can indirectly impair their functions. Oxidative stress caused by the T-2 toxin is by depleting antioxidant reserves and promoting lipid peroxidation, causing damage to the cellular membranes and proteins, including transporters like SGLT1 and SGLT2, in the kidneys. These transporters play a key role in reabsorbing glucose from the renal filtrate, and the oxidative damage could lead to a substantial impairment of their activity. Furthermore, the disruption of the protein synthesis that is caused by T-2 inhibits proper ribosomal function, which subsequently also reduces the synthesis of proteins that are required for transporter function and membrane integrity. This inhibition could diminish the expression or activity of SGLT1 and SGLT2 in the renal tubules [25,45]. Due to the oxidative stress and triggering inflammation and apoptosis, T-2 toxin can lead to tubular injury in the kidneys and this damage might compromise its ability to maintain proper glucose reabsorption, indirectly affecting SGLT1 and SGLT2 functions [46]. In a study by Holme, J.A. in 2003 [47], T-2 induced concentration-dependent apoptosis in HL-60 human promyelocytic leukemia cells after 24 h, starting already at concentrations of 3.1 and 6.25 ng/mL, respectively. An increased number of apoptotic cells was observed after exposure with 12.5 ng/mL of toxin after 4–6 h. After exposure to concentrations of toxins (25–50 ng/mL), little cytotoxicity (plasma membrane damage) was noted that induced apoptosis in 60–100% of the cells [48].

As the kidneys are among the main organs affected by T-2 mycotoxin and have an essential role in glucose homeostasis, and because the immunolocalization of sodium-dependent glucose transporters in the kidney tissue of birds has not yet been fully elucidated, our current study was carried out on the kidney material of domestic chickens; thus, we investigated the effect of T-2 on SGLT1 and SGLT2, which are members of the SLC5A gene family and contribute to renal glucose reabsorption [49]. Compared to the staining of SGLT1 in chicken kidney tissue, our study revealed a strong staining of SGLT2 in the control group and a significant reduction of the expression in the T-2 toxin group in the epithelial cells of the renal proximal tubules, presumably because SGLT2 is considered to be the main glucose transporter involved in glucose reabsorption in the kidneys; it carries out 80–90% of glomerular filtered glucose reabsorption in the renal tubular system [12,13]. The significant reduction from predominantly strong expression of SGLT2 in the control group to mainly weak expression in the T-2 toxin group could indicate that SGLT2, as the major glucose co-transporter, is affected more by the T-2 mycotoxin compared to SGLT1. With visual control, it could be observed that, compared to SGLT1, which was localized mainly in the apical region of the epithelial cells, SGLT2 was immunolocalized throughout the epithelial cells of the proximal tubules. In the T-2 toxin group, pale staining of the proximal renal tubules was observed; the weak expression of both of the studied antibodies and the morphological changes in the T-2-intoxicated bird group observed in our current study suggest that the intoxication caused a reduction in glucose transport. Furthermore, the irregular and damaged brush border membranes of the proximal tubule’s epithelial cells in the T-2-intoxicated chicken group were observed in our present study. This finding is also in accordance with that of previous studies showing that T-2 induces oxidative stress with detrimental effects, such as nuclear and mitochondrial DNA damage and disturbances in cell signaling, as the toxins can affect the cell cycle and lead to apoptosis and, ultimately, the death of the cells [49,50,51]. Specifically, some of the main T-2 toxic effects on the kidney are apoptosis of the proximal convoluted tubule’s epithelial cells, degeneration of the epithelial lining, swelling and vacuolar degeneration of the tubular epithelium, and necrosis of the renal tubules [25]. These pathological alterations indicate the nephrotoxicity of T-2 and are in accordance with the findings of our study. The pale staining of the proximal renal tubules in the T-2 toxin group of both major glucose transporters in the kidneys indicates a reduced ability to adequately carry out glucose transport; this is caused by T-2 mycotoxicosis and supports the hypothesis that there are morphological changes in the kidney tissue during intoxication. As the current primary research was limited with the comparative study of the immunohistochemical localization of the Na(+)-dependent glucose co-transporters SGLT1 and SGLT2 in chicken kidneys, to clarify the pathomorphological changes and specify the strength of the expression of the glucose transporters’ in the laying chicken’s kidney tissue during mycotoxicosis in greater detail, more studies using different research methods, such as immunofluorescence, Western blot, or mRNA quantification, should be carried out in the future.

## 5. Conclusions

This study revealed the immunolocalization of SGLT1 and SGLT2 in the kidney tissue of healthy and intoxicated young female chicks. The sodium-dependent glucose co-transporters SGLT1 and SGLT2 were localized in the epithelial cells of the renal proximal tubules. The expression of SGLT2 was observed to be comparatively stronger than the expression of SGLT1, which localized mainly in the apical membranes of the epithelial cells of the proximal tubules. In the comparison of the expression of the studied antibodies in the kidneys of the chickens of the two study groups, the expression of both antibodies was relatively weaker in the kidneys of the chicks belonging to the T-2 toxin group. Together with the morphological changes, i.e., the irregular and damaged brush border membranes of the proximal tubule’s epithelial cells, the weak expression of both major glucose transporters, SGLT1 and SGLT2, in the kidney tissue of the chickens in the T-2 toxin group may indicate kidney tissue damage and, subsequently, a reduced functionality of glucose transport in the kidneys during T-2 mycotoxicosis. The knowledge gained during this primary study is novel and necessary for further subsequent studies.

## Figures and Tables

**Table 1:** Expression of SGLT1 and SGLT2 in the proximal tubules of the chicken kidneys.

Antibody	Chicken nr.	Control Group	T-2 Toxin Group
SGLT1 *	1	++	+
	2	+++	++
	3	++	+
	4	++	+
	5	++	++
	6	++	+
	7	++	+
SGLT2 *	1	+++	+
	2	+++	++
	3	+++	++
	4	+++	+
	5	++	+
	6	+++	+
	7	+++	+

* The intensity of staining using eye visual control is shown as: + weak; ++ moderate; +++ strong.

**Table 2:** Mean intensity and statistical significance of SGLT1 and SGLT2 expression in healthy and T-2 toxin-treated chicken renal proximal tubules.

Group	SGLT1		SGLT2	
	Control Group	T-2 Toxin Group	Control Group	T-2 Toxin Group
Mean intensity	2.14	1.14	2.86	1.29
SD	0.40	0.41	0.42	0.54
Paired *t*-test *p*-value *	0.000203	0.000204	0.000014	0.000015
Wilcoxon signed-rank test *p*-value *	0.015625	0.015626	0.015627	0.015628

* *p* < 0.05.

## Data Availability

The original contributions presented in this study are included in the article material. Further inquiries can be directed to the corresponding author.

## References

[1] Hruby V.J., Bittar E., Bittar N. (1997). Molecular and Cellular Endocrinology.

[2] Mota M., Mota E., Dinu I.R., Croniger C. (2015). Treatment of Type 2 Diabetes.

[3] König H.E., Korbel R., Liebich H.G. (2016). Avian Anatomy Textbook and Colour Atlas.

[4] Vallon V., Thomson S.C. (2012). Renal Function in Diabetic Disease Models: The Tubular System in the Pathophysiology of the Diabetic Kidney. Annu. Rev. Physiol..

[5] Haas B., Eckstein N., Pfeifer V., Mayer P., Hass M.D.S. (2014). Efficacy, Safety and Regulatory Status of SGLT2 Inhibitors: Focus on Canagliflozin. Nutr. Diabetes.

[6] Mather A., Pollock C. (2011). Glucose Handling by the Kidney. Kidney Int..

[7] Takata K. (1996). Glucose Transporters in the Transepithelial Transport of Glucose. J. Electron Microsc..

[8] Sano R., Shinozaki Y., Ohta T. (2020). Sodium–Glucose Cotransporters: Functional Properties and Pharmaceutical Potential. J. Diabetes Investig..

[9] Navale A.M., Paranjape A.N. (2016). Glucose Transporters: Physiological and Pathological Roles. Biophys. Rev..

[10] Raja M., Puntheeranurak T., Hinterdorfer P., Kinne R., Mark O.B. (2012). SLC5 and SLC2 Transporters in Epithelia—Cellular Role and Molecular Mechanisms. Current Topics in Membranes.

[11] Allmang C., Hussar P., Järveots T., Duritis I. Sodium-glucose co-transporter SGLT2 in kidneys of chicken in different ages. Proceedings of the 21st Congress of the International Federation of Associations of Anatomists in conjunction with the 74th Annual Meeting of the Korean Association of Anatomists, Kimdaejung Convention Center.

[12] You G., Lee W.-S., Barros E.J.G., Kanai Y., Huo T.-L., Khawaja S., Wells R.G., Nigam S.K., Hediger M.A. (1995). Molecular Characteristics of NA+-Coupled Glucose Transporters in Adult and Embryonic Rat Kidney. J. Biol. Chem..

[13] Bonora B.M., Avogaro A., Fadini G.P. (2020). Extraglycemic Effects of SGLT2 Inhibitors: A Review of the Evidence. Diabetes Metab. Syndr. Obes..

[14] Horiba N., Masuda S., Takeuchi A., Takeuchi D., Okuda M., Inui K.-I. (2003). Cloning and Characterization of a Novel NA+-Dependent Glucose Transporter (NAGLT1) in Rat Kidney. J. Biol. Chem..

[15] Hussar P., Dūrītis I., Popovska-Percinic F., Järveots T. (2020). Short communication: Immunohistochemical study of sodium-dependent glucose co-transporters in ostriches kidneys. Agraarteadus J. Agric. Sci..

[16] Hussar P., Allmang C., Popovska-Percinic F., Järveots T., Dūrītis I. (2022). Comparative Study of Sodium-Dependent Glucose Co-Transporters in Kidneys of Ostrich Chickens. Sci. Hori..

[17] Hussar P., Kaerner M., Duritis I., Plivca A., Pendovski L., Jaerveots T., Popovska-Percinic F. (2017). Temporospatial Study of Hexose Transporters and Mucin in the Epithelial Cells of Chicken (*Gallus gallus domesticus*) Small Intestine. Pol. J. Vet. Sci..

[18] Varzakas T., Agriopoulou S., Stamatelopoulou E. (2024). Mycotoxin. Encyclopedia. https://encyclopedia.pub/entry/22939.

[19] Nazari L., Pattori E., Terzi V., Morcia C., Rossi V. (2014). Influence of Temperature on Infection, Growth, and Mycotoxin Production by Fusarium Langsethiae and F. Sporotrichioides in Durum Wheat. Food Microbiol..

[20] Nathanail A.V., Varga E., Meng-Reiterer J., Bueschl C., Michlmayr H., Malachova A., Fruhmann P., Jestoi M., Peltonen K., Adam G. (2015). Metabolism of the Fusarium Mycotoxins T-2 Toxin and HT-2 Toxin in Wheat. J. Agric. Food. Chem..

[21] Nayakwadi S., Ramu R., Sharma A.K., Gupta V.K., Rajukumar K., Kumar V., Shirahatti P.S., Rashmi L., Basalingappa K.M. (2020). Toxicopathological Studies on the Effects of T-2 Mycotoxin and Their Interaction in Juvenile Goats. PLoS ONE.

[22] Edwards S.G., Imathiu S.M., Ray R.V., Back M., Hare M.C. (2012). Molecular Studies to Identify the Fusarium Species Responsible for HT-2 and T-2 Mycotoxins in UK Oats. Int. J. Food Microbiol..

[23] Lippolis V., Pascale M., Maragos C.M., Visconti A. (2008). Improvement of Detection Sensitivity of T-2 and HT-2 Toxins Using Different Fluorescent Labeling Reagents by High-Performance Liquid Chromatography. Talanta.

[24] Kiš M., Vulić A., Kudumija N., Šarkanj B., Tkalec V.J., Aladić K., Škrivanko M., Furmeg S., Pleadin J. (2021). A Two-Year Occurrence of Fusarium T-2 and HT-2 Toxin in Croatian Cereals Relative of the Regional Weather. Toxins.

[25] Janik E., Niemcewicz M., Podogrocki M., Ceremuga M., Stela M., Bijak M. (2021). T-2 Toxin—The Most Toxic Trichothecene Mycotoxin: Metabolism, Toxicity, and Decontamination Strategies. Molecules.

[26] Male D., Wu W., Mitchell N.J., Bursian S., Pestka J.J., Wu F. (2016). Modeling the Emetic Potencies of Food-Borne Trichothecenes by Benchmark Dose Methodology. Food Chem. Toxicol..

[27] Pinton P., Oswald I. (2014). Effect of Deoxynivalenol and Other Type B Trichothecenes on the Intestine: A Review. Toxins.

[28] Chi M.S., Mirocha C.J., Kurtz H.J., Weaver G., Bates F., Shimoda W. (1977). Effects of T-2 Toxin on Reproductive Performance and Health of Laying Hens. Poult. Sci..

[29] Henghold W.B. (2004). Other biologic toxin bioweapons: Ricin, staphylococcal enterotoxin B, and trichothecene mycotoxins. Dermatol Clin..

[30] Afsah-Hejri L., Jinap S., Hajeb P., Radu S., Shakibazadeh S. (2013). A Review on Mycotoxins in Food and Feed: Malaysia Case Study. Compr. Rev. Food Sci. Food Saf..

[31] Cope R.B., Gupta R.C. (2018). Veterinary Toxicology.

[32] Lancova K., Hajslova J., Poustka J., Krplova A., Zachariasova M., Dostalek P., Sachambula L. (2008). Transfer ofFusariummycotoxins and ‘Masked’ Deoxynivalenol (Deoxynivalenol-3-Glucoside) from Field Barley through Malt to Beer. Ood Addit. Contam.-Chem. Anal. Control Expo. Risk Assess..

[33] Foroud N.A., Baines D., Gagkaeva T.Y., Thakor N., Badea A., Steiner B., Bürstmayr M., Bürstmayr H. (2019). Trichothecenes in Cereal Grains—An Update. Toxins.

[34] He J., Zhou T., Young J.C., Boland G.J., Scott P.M. (2010). Chemical and Biological Transformations for Detoxification of Trichothecene Mycotoxins in Human and Animal Food Chains: A Review. Trends Food Sci. Technol..

[35] Meneely J., Greer B., Kolawole O., Elliott C. (2023). T-2 and HT-2 Toxins: Toxicity, Occurrence and Analysis: A Review. Toxins.

[36] Carson F.L. (1997). Histotechnology: A Self-Instructional Text.

[37] Deepa K.P., Sreeranjini A.R., Soumya C.B., Mayadany S., Sunilkumar N.S., Sumena K.B. (2021). Comparative histological studies on the renal medulla in broiler chicken and broiler duck. Int. J. Vet. Sci. Anim. Husb..

[38] Boron W.F., Boulpaep E.L. (2016). Medical Physiology.

[39] Triplitt C.L. (2012). Understanding the kidneys’ role in blood glucose regulation. Am. J. Manag. Care.

[40] Allmang C., Hussar P., Duritis I., Järveots T. (2024). Immunolocalization of sodium-dependent glucose co-transporter 1 and sodium-dependent glucose co-transporter 2 in chicken’s (*Gallus gallus domesticus*) kidneys. Pol. J. Vet. Sci..

[41] Bhatnagar D., Yu J., Ehrlich K.C. (2002). Toxins of filamentous fungi. Chem Immunol..

[42] Zain M.E. (2011). Impact of mycotoxins on humans and animals. J. Saudi Chem. Soc..

[43] Türker L., Gümüş S. (2009). A Theoretical Study on Vomitoxin and Its Tautomers. J. Hazard. Mater..

[44] Devreese M., De Backer P., Croubels S. (2013). Verschillende Methoden Om Mycotoxineproductie En de Impact Op de Diergezondheid Tegen Te Gaan. Vlaams Diergeneeskd. Tijdschrift..

[45] Adhikari M., Negi B., Kaushik N., Adhikari A., Al-Khedhairy A.A., Kaushik N.K., Choi E.H. (2017). T-2 mycotoxin: Toxicological effects and decontamination strategies. Oncotarget.

[46] Stoev S.D., Diakov L., Koynarski V., Angelov A. (2010). Special Pathology and Diagnostics of Mycoses, Mycotoxicoses, Parasitoses, Intoxications and Avitaminoses.

[47] Holme J.A., Morrison E., Samuelsen J.T., Wiger R., Låg M., Schwarze P.E., Bernhoft A., Refsnes M. (2003). Mechanisms involved in the induction of apoptosis by T-2 and HT-2 toxins in HL-60 human promyelocytic leukemia cells. Cell Biol Toxicol..

[48] Wright E.M. (2001). Renal Na+-Glucose Cotransporters. Am. J. Physiol..

[49] Sudakin D.L. (2003). Trichothecenes in the Environment: Relevance to Human Health. Toxicol. Lett..

[50] Ueno Y. (1984). Toxicological Features of T-2 Toxin and Related Trichothecenes. Fundam. Appl. Toxicol..

[51] Wu Q.-H., Wang X., Yang W., Nüssler A.K., Xiong L.-Y., Kuča K., Dohnal V., Zhang X.-J., Yuan Z.-H. (2014). Oxidative Stress-Mediated Cytotoxicity and Metabolism of T-2 Toxin and Deoxynivalenol in Animals and Humans: An Update. Arch. Toxicol..

